# REPLACR-mutagenesis, a one-step method for site-directed mutagenesis by recombineering

**DOI:** 10.1038/srep19121

**Published:** 2016-01-11

**Authors:** Ashutosh Trehan, Michał Kiełbus, Jakub Czapinski, Andrzej Stepulak, Ilpo Huhtaniemi, Adolfo Rivero-Müller

**Affiliations:** 1 Department of Physiology, Institute of Biomedicine, University of Turku, Turku, Finland; 2 Department of Surgery and Cancer, Institute of Reproductive and Developmental Biology, Hammersmith Campus, Imperial College London, London, United Kingdom; 3 Faculty of Natural Sciences and Technology, Åbo Akademi University, Turku, Finland; 4 Department of Biochemistry and Molecular Biology, Medical University of Lublin, Lublin, Poland

## Abstract

Mutagenesis is an important tool to study gene regulation, model disease-causing mutations and for functional characterisation of proteins. Most of the current methods for mutagenesis involve multiple step procedures. One of the most accurate methods for genetically altering DNA is *recombineering*, which uses bacteria expressing viral recombination proteins. Recently, the use of *in vitro* seamless assembly systems using purified enzymes for multiple-fragment cloning as well as mutagenesis is gaining ground. Although these *in vitro* isothermal reactions are useful when cloning multiple fragments, for site-directed mutagenesis it is unnecessary. Moreover, the use of purified enzymes *in vitro* is not only expensive but also more inaccurate than the high-fidelity recombination inside bacteria. Here we present a single-step method, named REPLACR-mutagenesis (*R*ecombineering of *E*nds of linearised *PLA*smids after P*CR*), for creating mutations (deletions, substitutions and additions) in plasmids by *in vivo* recombineering. REPLACR-mutagenesis only involves transformation of PCR products in bacteria expressing Red/ET recombineering proteins. Modifications in a variety of plasmids up to bacterial artificial chromosomes (BACs; 144 kb deletion) have been achieved by this method. The presented method is more robust, involves fewer steps and is cost-efficient.

Site-directed mutagenesis (SDM), also known as directed mutagenesis, is used to generate mutations, add or delete domains in cDNAs or gene promoters in order to study the resulting translation product for protein engineering and/or functional characterisation. SDM is also used to model mutations found in clinical samples for functional studies at the cellular and molecular level.

There are a vast number of techniques and commercial kits to generate point mutations, short additions or deletions, mainly based on PCR such as overlap extension and megaprimer PCR^1^,^2^,^3^,^4^,^5^,^6^,^7^. Yet most involve several steps and are limited by the size of insertion or deletion due to the use of complementary primer pairs. In most cases, mutagenesis is accompanied with ligation of the resulting linear PCR fragment to form a circular vector, which is inefficient and can result in unwanted additions or deletions due to the activities of the polymerase used.

Homologous recombination (HR) is a process where a pair of homologous DNA fragments exchanges nucleotides, often as means to repair DNA breaks. HR was first used to modify genetic material in yeast^8^. Later on, the bacteriophage enzymes that perform HR independently of the bacterial system were discovered and used to modify plasmids, bacterial artificial chromosomes (BACs), and bacterial genomes^9^,^10^,^11^,^12^, a method now called *recombineering* (recombination-mediated genetic engineering)^13^. These enzymes allow genetic modifications by recognising complementary DNA strands for strand invasion or annealing^14^,^15^. One limitation for this seamless cloning technique is the need of selection of positive clones, something we, among many others, have solved by using selection/counter-selection systems^16^,^17^,^18^,^19^.

Recently, a series of methods based on the assembly of overlapping DNA fragments using *in vitro* reactions have been developed, such as Gibson assembly^20^, GeneArt seamless cloning (Life technologies), In-Fusion HD cloning (Clontech), ligase-independent cloning (LIC) and the variations of the latter (SLIC and SLICE)^21^,^22^,^23^. These *in vitro* methods can join several fragments of DNA with 15–25 nucleotide homology at both termini. Yet, there are limitations for these assemblies such as single-stranded DNA secondary structures and cloning of primer dimers^24^. A common feature of these methods is the use of purified DNA-modifying enzymes (exonucleases, recombinases, polymerases and ligases), which not only makes these methods highly expensive but also dependent on selective buffers and reaction conditions. In addition, *in vitro* DNA termini-joining can also yield many non-specific products, which is in stark contrast to *in vivo* recombineering that is highly selective and robust due to the presence of endogenous proofreading replication and repair pathways.

Although *in vitro* methods are highly advantageous for multiple fragment cloning, they are, however, completely unnecessary for generating mutations at a single locus (substitutions, additions or deletions) in plasmids. Here we present a single-step method where, by *in vivo* recombineering, we are able to generate site-directed modifications (point mutations, deletions, additions and substitutions) in plasmid vectors in a cost-effective manner with high efficiency and accuracy. Since the method is primarily focused on generating mutations in plasmids and the only step needed is PCR followed by recombineering of the linearised ends of the plasmid, we named the method as REPLACR-mutagenesis (*R*ecombineering of *E*nds of linearised *PLA*smids after P*CR*; Fig. 1).

## Results

### Optimal homology for REPLACR-mutagenesis

We rationalised that the linear ends of a PCR product could be circularised by *recombineering* if they had enough homology. Thus, we first amplified a plasmid by PCR such that the resulting linear PCR products had a varying number of homologous nucleotides at their ends. To determine the optimal length of homology needed for highest recombination efficiency, we tested 23 bp primer pairs with homology ranging from 2 bp to 23 bp (See Supplementary Table S1 for primer sequences). The primers targeted a *ScaI* restriction site (AGTACT) in the wild-type (WT) human luteinizing hormone/chorionic gonadotropin receptor (*LHCGR*) plasmid^25^ by the addition of two nucleotides (AT) in the middle of a *ScaI* sequence, such that the resulting site in the plasmid was not amenable to *ScaI* digestion. The number of correct clones found over the LHCGR_WT background was used to calculate the efficiency with respect to the homology at the ends of the PCR products (Fig. 2a). A 2 bp homology at the ends of PCR products is insufficient for recombination, thereby resulting in no positive clones. From 5 bp homology onwards, there was an increase in efficiency of REPLACR-mutagenesis up to 17 bp, with the maximum efficiency of 84%. Since the primers were only 23 bp long, a 20 bp and 23 bp homology among the primers favoured the formation of primer dimers thereby resulting in undesired PCR products. The bacterial colonies containing either the mutated plasmids or LHCGR_WT background were screened by colony PCR and subsequent *ScaI* restriction digestion of the PCR products. Supplementary Figure S1 shows the *ScaI* restriction digestion patterns of PCR products obtained for one representative experiment, and the results are summarised in Supplementary Table S2, detailing the number of colonies obtained, screened, correct ones found and the associated efficiencies. The colony PCR conditions are mentioned in Supplementary Table S3.

Using oligonucleotides that span 22–24 nucleotides of primer-template binding plus a 5′-tail containing the homology arms can further increase the total length of the homology. As mentioned later in the manuscript, different mutants were made where longer primers with more more than 17 bp homology (20, 23 and 30 bp homology) were used, however, the associated efficiencies were very similar to the 84% efficiency as achieved by 17 bp homology (See Supplementary Figure S2). Thus, a 17 bp homology with 3′ overhangs for both primers is sufficient for efficient REPLACR-mutagenesis.

In addition, when three PCR products with 11 bp, 14 bp and 17 bp homology were transformed in non-*recombineering* bacteria (*E.coli* DH-10β), no colonies were obtained because linear PCR products cannot recombine own their own in the absence of enzymes needed for recombination.

### Efficiency comparison with commercial kits (Gibson Assembly and GeneArt seamless cloning)

Since the PCR products with 14 bp and 17 bp homology at their termini gave highest efficiencies with REPLACR-mutagenesis (Fig. 2a), we used the same PCR products with two commercially available kits, namely Gibson assembly and GeneArt seamless cloning. The efficiencies of the Gibson assembly and GeneArt seamless cloning were similarly determined by *ScaI* digestion of the colony PCR products (see Supplementary Fig. S3). Although PCR products with 14 bp homology gave higher efficiencies with Gibson assembly and GeneArt, but for PCR products with recommended 17 bp homology, efficiencies all the methods were comparable (Fig. 2b). Moreover, we consistently found GeneArt cloning to result in smaller number of overall colonies as compared with REPLACR-mutagenesis or Gibson Assembly (see Supplementary Table S4).

### Substitutions

All substitutions were targeted to plasmids encoding the human *LHCGR*, follicle-stimulating hormone receptor (*FSHR*) or beta-2 adrenergic receptor (*β2AR*). We generated many single nucleotide substitutions, namely, LHCGR_Asn291Ser, LHCGR_Val454Ile, FSHR_Ala444Thr, FSHR_ Gly70Ala, β2AR_Asp79Asn, β2AR_Asp130Asn and β2AR_Cys341Gly and a double nucleotide substitution, β2AR_Tyr350Ala (See Supplementary Figure S4). The primers used for creating substitutions and verifying via DNA sequencing are mentioned in Supplementary Tables S5 and S6, respectively.

### Deletions

Because the method is not limited to point substitutions, we also tested whether we could delete nucleotides. First, we deleted a single nucleotide in the *LHCGR* (1850delG), which results in a frame-shift of the *C-*terminal tail of the receptor; the functional tests for this mutant receptor have been reported elsewhere^26^. This deletion was achieved as verified by sequencing (see Supplementary Figure S5). As deletion of a nucleotide worked as efficiently as nucleotide substitutions, we proceeded to generate larger editing of the DNA sequence of some plasmids.

The deletion of 12 nucleotides of the signal peptide of the *LHCGR* gene (LHCGR-Lys12-Leu15del) was achieved using the same method. Once again, we produced hundreds of colonies where most of them were correct as verified by sequencing (see Supplementary Figure S5).

Finally, we used REPLACR-mutagenesis to delete an entire 144 kb DNA sequence from a human *LHCGR* BAC clone (RPCI-11-186L7), a one step “BAC-shaving” as compared with the multistep systems to-date^27^. The primer sequences used for the deletion are mentioned in Supplementary Table S5. The resulting deletion was verified by sequencing (Supplementary Fig. S5). The primers used for sequencing are specified in Supplementary Table S6. PCR conditions for deletion are mentioned in Supplementary Table S7. In addition, since the chloramphenicol resistance gene and origin of replication were not affected during PCR, the resulting plasmid could propagate in chloramphenicol containing Luria-Bertani (LB) medium, demonstrating the integrity of the backbone during PCR.

### Additions

In order to generate mutants with additional nucleotides, the LHCGR_Leu10-Gln17Dup, a 27-nucleotide duplication, was generated. This construct produced many colonies but most of them were negative, probably due to a preference for near-end recombination and the presence of 2 identical sequences (duplication). The correct colonies were obtained by generating forward and reverse strands separately in two separate PCR reactions and thereafter the two products were mixed together. The PCR products were heated up to 95 °C and slowly allowed to cool for annealing of DNA strands, followed by *DpnI* digestion before transformation in Red/ET bacteria, as previously described^28^. The sequence was verified by sequencing (Supplementary Figure S6). Neither Gibson assembly nor GeneArt produced any correct colonies for this duplication.

In order to show that there is no limit on the number of additional nucleotides to be incorporated as far as they have homology arms, we inserted a 45-nucleotide nuclear localization signal to a plasmid containing Cryptochrome Circadian Clock 2 (CRY2) gene^29^. Moreover, a 60-nucleotide addition of a flexible domain was also achieved using REPLACR-mutagenesis (Supplementary Figure S6). The primer sequences used to generate the mutations are mentioned in Supplementary Tables S5. The sequencing primers used to verify the additions of 45 and 60 nucleotides were TTGCTCGTTGGCATCAGAAGG and AGCTGCTGCTAATGCAGGAT, respectively. We did not try longer additions as the cost for larger primers become higher than using synthetic DNA “blocks” and Gibson assembly.

### Modifications of larger plasmids

A 24 kb *Wnt1* targeting vector was used to introduce a 2 bp addition. The two bases (TG) were introduced in one the three *MfeI* restriction sites (CAATTG) present in the original vector such that the resulting sequence (CAATGTTG) leaves only two *MfeI* restriction sites in the mutated plasmid. *MfeI* restriction digestion of the original *Wnt1* vector with three *MfeI* restriction sites results in three bands (15476, 6379, 1844 bp; lane 2 in Fig. 3) whereas the modified *Wnt1* vector with a mutated *MfeI* site results in two bands (15476 and 8225 bp; lane 4 in Fig. 3), as expected. The mutated region was verified by sequencing (Supplementary Figure S7). The primers for creating the mutation and sequence verification by DNA sequencing are stated in Supplementary Tables S5 and S6, respectively. The PCR conditions are mentioned in Supplementary Table S8. The mutated plasmid could replicate in kanamycin conditioned LB-agar plates, similar to the original plasmid, demonstrating the integrity of the antibiotic resistance gene as well as the origin of replication. Overall, the sequencing of the mutated site, the expected restriction digestion pattern and the ability of the plasmid to propagate in bacteria cultured in the appropriate antibiotic containing medium demonstrates the integrity of a larger and complex plasmid mutated with REPLACR-mutagenesis.

### Modifying plasmid with similar incompatibility to the recombineering plasmid

A potential limitation of REPLACR-mutagenesis could be the modification of plasmids with origin of replication incompatible with the Red/ET plasmid (pSC101). To test this, *recombineering* bacteria were prepared with Red/ET plasmid carrying tetracycline resistance gene. We have previously modified a similar Red/ET plasmid (pSC101 BADgbaRecA) containing hygromycin rather than tetracycline resistance gene^16^. A fragment of the temperature sensitive repressor (RepA) was deleted (944 bp) and was verified by sequencing (Supplementary Figure S8). The primers for mutagenesis, sequencing and the PCR conditions are specified in Supplementary Tables S5, S6 and S9, respectively. The original plasmid can only be grown at 30 °C due to active repressor while the mutated plasmid with an inactive repressor could now be grown at 37 °C. This shows the utility of the REPLACR-mutagenesis to modify plasmids with similar incompatibility to the recombineering plasmid. This is possible because the electrocompetent bacteria are already expressing the recombination enzymes during their preparation and the original recombineering plasmid cannot replicate at 37 °C, thereby eliminating any selective pressure. However, we found very few colonies after REPLACR-mutagenesis and the recombination efficiency of 33% as only one out three colonies were positive. This suggests that the incompatibility of the origins of replication has a negative effect, which result in low efficiencies, but it is possible to achieve mutations even in such circumstances.

## Discussion

**Figure 1:**
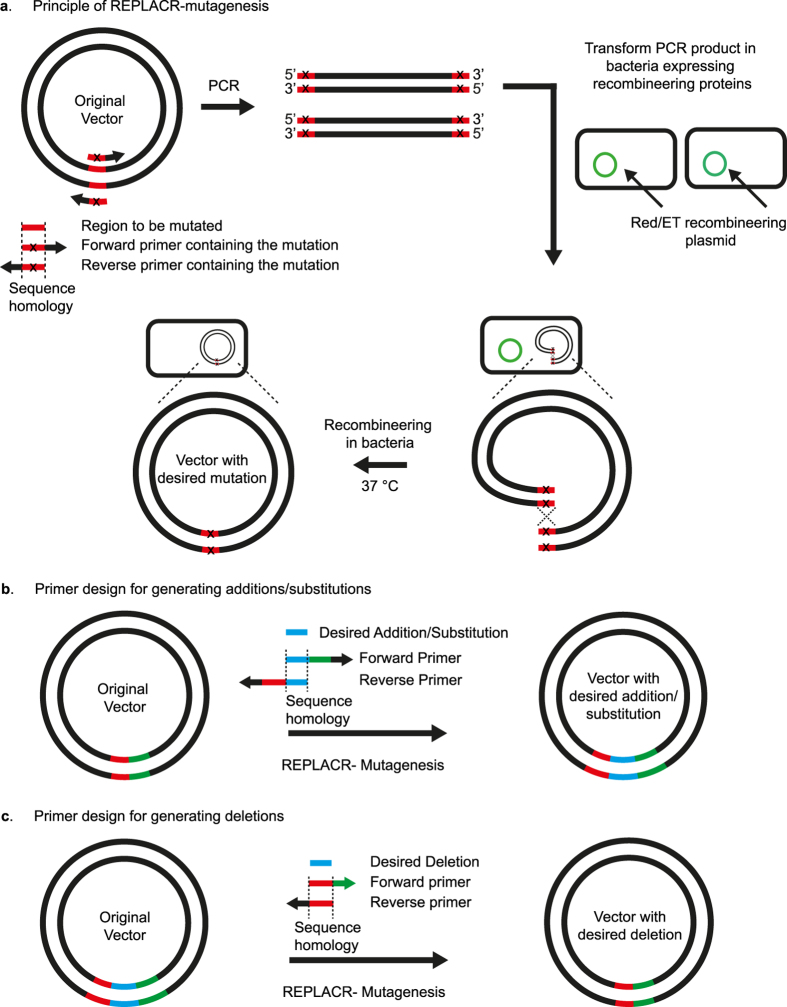
Principle of REPLACR-mutagenesis and primer design strategy for sequence substitution, addition or deletion. **(a)** Primers containing the desired mutation are designed to target a specific region in the original vector. A high-fidelity polymerase is used to generate a linear PCR product such that both the ends contain overlapping sequences for recombination. Bacteria expressing the *recombineering* proteins (Redγ, β, α and RecA) are transformed with the PCR product. Recombination takes places inside the bacteria thereby yielding a circular plasmid containing the desired mutation. Bacterial colonies are then screened for the correct clone by PCR and sequencing. **(b)** Forward and reverse primers contain the desired addition/substitution as a part of homology regions needed for recombination, besides containing a 3′ extension for effective template binding. The homology region (17 bp or more) for substitutions also contains the desired nucleotide change. **(c)** For generating deletion mutants, forward primer contains the sequence adjoining the sequence to be deleted. The reverse primer however contains a sequence homologous to the forward primer and the adjoining sequence in the vector.

**Figure 2:**
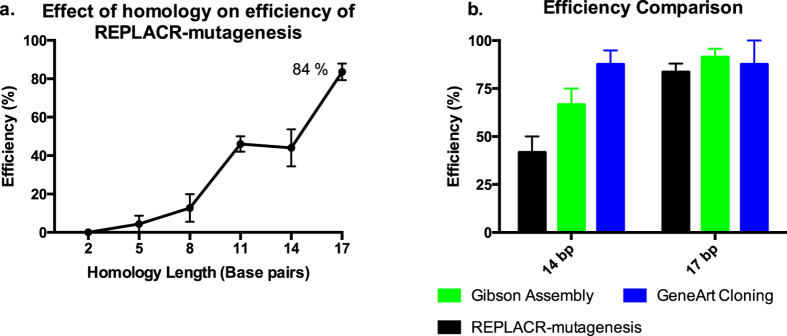
Efficiency of REPLACR-mutagenesis. (**a**) The effect of homology at the ends of PCR products is plotted against the achieved efficiencies with REPLACR-mutagenesis. The efficiency of mutagenesis increases with increasing homology, where a 2 bp homology is insufficient to yield any correct products while a 17 bp homology gives the highest efficiency at 84%. (**b**) The same PCR products with 14 bp and 17 bp homology were used with two commercial kits (Gibson Assembly and GeneArt Seamless cloning) and the achieved efficiencies were compared with REPLACR-mutagenesis. REPLACR-mutagenesis is least efficient among the three methods when the PCR products have only 14 bp homology, however with the recommended 17 bp homology at the ends of PCR products, comparable efficiencies for all the methods can be observed. The data is presented as mean ± standard error of mean (SEM) of three independent repeats.

**Figure 3:**
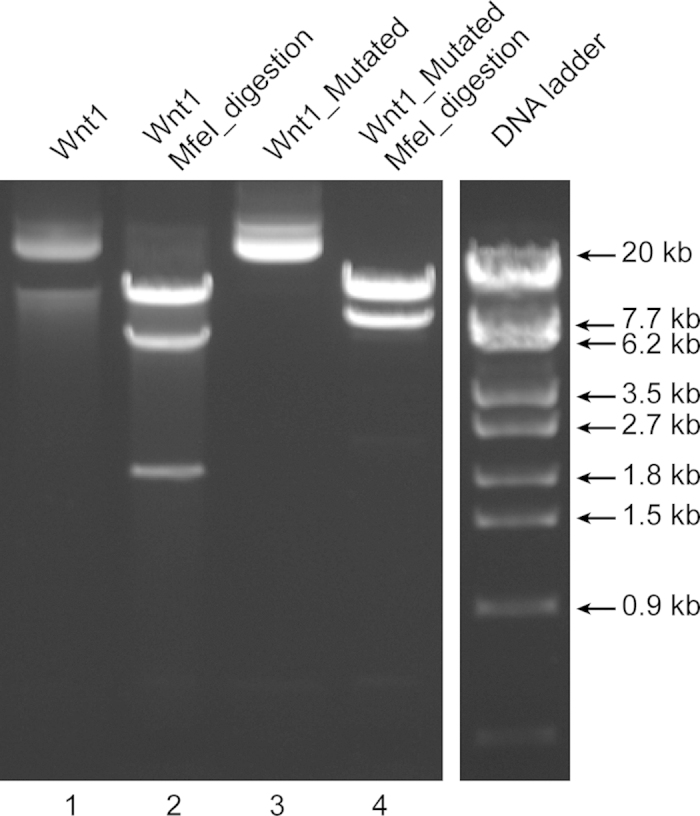
Modifications in larger plasmids. The 24 kb *Wnt1* targeting vector (undigested; lane 1) contains three *MfeI* restriction sites, which upon *MfeI* digestion gives three bands (15476, 6379, 1844 bp; lane 2). One of the *MfeI* sites is mutated by REPLACR-mutagenesis, such that the resulting plasmid (lane 3) upon *MfeI* digestion gives only two bands (15476 and 8225 bp; lane 4).

REPLACR-mutagenesis presents a quick and robust *in vivo* recombineering based mutagenesis protocol. The only step needed is the transformation of PCR products in bacteria expressing viral recombination proteins. An effective primer design is thus crucial for this method. The general primer design strategy, specific for additions, deletions and substitutions is summarised in Fig. 1. Primers should be designed such that the resulting PCR products contain a homology at their termini of around 17bp. As seen in Fig. 2a, a 17 bp homology at the ends of PCR products gave the highest efficiency of 84% over the background. For 23 bp primer pairs, up to 17 bp homology (with 6 additional nucleotides at 3′ end) resulted in expected PCR products whereas a 20 bp and 23 bp homology among primers resulted in undesired PCR products. Therefore, besides containing the homology regions, the primers should include a 3′ extension (6 nucleotides or more), such that primer-template binding is favoured over primer-primer self-complementarity. For creating substitutions, the homology region also contains the substituted nucleotide(s), with an extended 3′ end. For additions, a 20 bp region for primer template binding is sufficient and the additions can be made on the 5′ end of either one or both primers. The added regions should contain a 17 bp homology for maximum efficiency. However, for creating deletions, one of the primers should contain the adjoining sequences between the region to be deleted and the other primer containing a 17 bp homology to the 5′ end of the first primer and an additional 3′ sequence in the other direction. It is however possible to design longer primers, with more than 17 bp homology but there is no significant increase in efficiency (see Supplementary Figure S2). In addition, longer primers also increase the cost as well as the chances of formation of secondary structures, thereby decreasing the PCR efficiency in some cases.

The bacteria used for recombination contain Red/ET plasmid, which during their preparation are made to express viral recombination proteins under arabinose promoter, by addition of *L*-Arabinose and subsequently frozen until use. The replication of Red/ET plasmid is temperature sensitive and only replicates at 30 °C^30^. Thus, after transforming the PCR products, the bacteria are grown at 37 °C and the resulting bacterial colonies only contain the desired mutated plasmid and not the original Red/ET plasmid. In addition, it is also possible to modify plasmids with incompatibility as the Red/ET plasmid because the electrocompetent bacteria are already expressing the recombination proteins to circularise the PCR products. However, the plasmid to be mutated should have a different antibiotic resistance gene than the Red/ET plasmid.

The method was used to successfully generate a variety of point substitutions, deletions ranging from one nucleotide deletion to as large as 144 kb deletion (shaving) in human *LHCGR* BAC in one single step, which would otherwise require multiple steps via traditional restriction digestion based deletion techniques or those involving selection/counter-selection cassettes^17^,^27^,^31^,^32^. We were also able to add nucleotides ranging from one to 60 nucleotides. The limitation of adding nucleotides by longer primers is the cost of primers themselves. The method was thus used to generate mutations in small plasmids (6–10 kb) to as large as a 24 kb *Wnt1* targeting vector.

Traditional PCR based mutagenesis methods typically require a variety of steps and the application of many enzymes such as kinases for phosphorylation of 3′ ends and ligases to form circular plasmids. Similarly, recombination-based mutagenesis and cloning methods (Gibson Assembly and GeneArt seamless cloning) also require the application of expensive enzymes and multiple steps. In both GeneArt and Gibson assembly, PCR of the template DNA is followed by DNA purification, *in vitro* recombination and subsequent transformation into bacteria. Our method reduces the number of steps needed for creating mutations to just one-step since the PCR product is directly transformed in the bacteria and there is no need for an additional *in vitro* incubation of PCR product with recombineering enzymes. In addition, it is considerably cheaper since the electrocompetent bacteria have to be prepared only once for a large number of mutagenesis experiments. Moreover, REPLACR-mutagenesis is as efficient as the commercially available Gibson assembly and GeneArt seamless cloning kits. Finally, a median efficiency of 75% was found for all the mutations made using our method (see Table 1).

The method bears similarity in primer design to PCR-based mutagenesis methods like quikchange site-directed mutagenesis (Agilent Technologies), where nicks in circular PCR products are repaired by bacterial endogenous DNA repair machinery^7^. However, most PCR products are linear and the number of circular PCR products with nicks that can be repaired by bacterial endogenous repair systems is very low, and henceforth the method becomes inefficient particularly for mutagenesis of more than one nucleotide. Thus, the expression of viral recombination proteins in bacteria as proposed in our method greatly enhances the efficiency of the recombination at the ends of linear PCR products as well as the number of bacterial colonies obtained. Moreover, REPLACR-mutagenesis is capable of complex additions and deletions in fewer steps than what would be needed by other PCR-based mutagenesis methods such as overlap extension PCR mutagenesis. Although there have been reports of using *in vivo* recombineering-based methods for mutagenesis in one single transformation step such as “*en passant mutagenesis”* but the bacterial colonies have to be grown and selected by colony PCR twice in different conditions, thereby prolonging the experiment^33^. The presented method however, involves direct screening of bacterial colonies obtained after the transformation step.

Although in the case of the large duplications we experienced problems during the PCR step, we solved it by generating single complementary strands of the vector and then re-joining them in an isothermal reaction, as previously described^28^, before transformation into Red/ET electrocompetent bacteria. The generation of complimentary DNA strands using two PCR reactions with only one primer each is a known way to PCR the DNA regions with tandem repeats. One of the limiting factors in REPLACR-mutagenesis is the PCR itself; since most high-fidelity polymerases are recommended for PCR products up to 20–25 kb, though there have been some improvements in development of better polymerases. Nevertheless, most plasmids containing the cDNA of genes are smaller than 10–12 kb and hence the utility of the method is sufficient for most routine mutagenesis experiments.

In conclusion, REPLACR-mutagenesis provides a cost-effective way involving fewer steps to produce mutations with high accuracy due to the nature of the Red/ET recombineering system.

## Methods

### Materials

KOD-Xtreme hot-start DNA polymerase was purchased from Merck Millipore. Three previously described plasmids carrying cDNA of the human *LHCGR*, *FSHR* and *β2AR* were used for all the substitutions^25^,^34^,^35^. Restriction endonucleases *DpnI* and *MfeI* were purchased from New England Biolabs (NEB) and *ScaI* was purchased from Promega. *Wnt1* targeting vector (24 kb) was purchased from the KOMP Repository (University of California Davis and Children’s Hospital Oakland Research Institute, USA). p*CRY2FL*(deltaNLS)-*mCherry*N1 was a gift from Chandra Tucker (Addgene plasmid # 26871)^29^. The Red/ET plasmid, pSC101BADgbaRecA[tet], was purchased from Genebridges (Dresden, Germany).

### Preparation of Electrocompetent bacteria for recombineering

Red/ET recombineering system (Red γ, β, α and RecA) containing recombineering plasmid (pSC101BADgbaRecA[tet]; hereafter called as Red/ET) was purchased from GeneBridges. Electrocompetent cells were prepared as described in the manuals from Genebridges using *L*-arabinose to induce the phage recombinases. Recombineering was performed in electrocompetent HS996 *E.coli* cells harbouring the Red/ET plasmid using standard recombination procedures^14^. Briefly, four microcentrifuge tubes with 1ml Luria-Bertani (LB) medium supplemented with either tetracycline (3 μg/ml) or hygromycin (15 μg/ml) were inoculated with bacteria harbouring the Red/ET plasmid and cultured overnight at 30 °C in a table-top thermomixer (Eppendorf). Next day, the 4 ml bacterial culture was transferred to a 250 ml LB culture with the appropriate antibiotic and was cultured for a further 3 h (250 rpm at 30 °C). Thereafter, *L*-arabinose was added to a final concentration of 0.35% and the temperature increased to 37 °C to induce the expression of the recombinases for 1 h. Bacteria were then collected by centrifugation 6000 *X g* for 15 min at 4 °C and were then resuspended in ice-cold water. This procedure was repeated once before resuspension in 10% glycerol, followed by centrifugation as above and resuspension of the bacteria in the remaining 10% glycerol (about 1 ml). Bacteria were then aliquoted into ice-cold microcentrifuge tubes (50 μl per tube) and snap-frozen in liquid nitrogen before storage at −80 °C until further use.

### PCR

PCR primers were designed to generate addition(s), substitution(s) or deletion(s) of specific regions in the wild-type (WT) *LHCGR*, *FSHR*, *β2AR* or *CRY2* plasmids^25^,^29^,^35^,^36^ (Fig. 1). Primer sequences specific for the mutation are listed in Supplementary Table S5. PCR was performed using a high-fidelity polymerase (KOD-Xtreme, Millipore) (see Supplementary Tables S7-S12 for PCR conditions). PCR products were purified by ethanol precipitation followed by *DpnI* digestion of the template plasmid. *DpnI*-digested PCR products were ethanol-precipitated and subsequently used for bacterial transformation. *DpnI* digestion can also be directly performed on the PCR products (1–2 μl) without purification and the *DpnI* digested products can then be transformed in recombineering bacteria.

### Site-directed mutagenesis

Mutagenesis involves only one-step: transformation of the PCR products generated using mutagenesis primers in recombineering bacteria (see Fig. 1 for principle and primer design).

### Recombineering

For generating mutants, one tube of electrocompetent cells was used per sample. Cells were first thawed on ice and the PCR product (100 ng) was then added, followed by electroporation in a 1-mm cuvette. Electroporation was performed at 1.35 kV, 25 μF, 200 ohms using an Eppendorf electroporator (2510). After electroporation, bacteria were incubated in 1 ml LB medium at 37 °C, shaking for 1–2 h before plating on LB-agar plates conditioned with the appropriate antibiotic(s).

Analysis of correct clones was performed first by PCR by primers flanking the targeted area using Biotools DNA polymerase and buffer. The general conditions included an initial denaturation at 96 °C for 2 min, followed by 30 cycles with 95 °C for 45 s, 57 °C for 45 s and 72 °C for 1–3 min, depending on the length of the product. Products were analysed by gel electrophoresis and then by sequencing (Turku Centre for Biotechnology, Finland). Sequencing was performed in both directions to ensure accuracy of the mutated modified sequences (See Supplementary Table S6 for sequencing primers).

### Determining optimal homology length needed for REPLACR-mutagenesis

Primers were designed for the addition of two nucleotides (AT) in the middle of a *ScaI* restriction site (AGTACT) in WT human *LHCGR* plasmid, thereby disrupting the restriction site^25^. The homology between forward and reverse primers was varied from 2bp to 23 bp (See Supplementary Table S1 for primer sequences). PCR products were subjected to REPLACR-mutagenesis protocol as mentioned above (Fig. 1). The resulting bacterial colonies were analysed by colony PCR (forward primer: AGGGTCCTGATTTGGCTGAT, reverse primer: TGGCATGTCTTAATCGCAGC; see Supplementary Table S3 for PCR conditions). The expected PCR product for the mutated plasmids should be 366 bp and not amenable to *ScaI* digestion whereas the LHCGR_WT background PCR product should be 364 bp and following *ScaI* digestion to yield 190 bp and 174 bp products (indistinguishable as a single band; see Supplementary Fig. S1).

### Gibson Assembly and GeneArt seamless cloning

The same PCR products with 14 bp and 17 bp homology, as used above with REPLACR-mutagenesis, were subjected to recombination by Gibson Assembly cloning (NEB) and GeneArt seamless cloning (Life technologies) kits following the manufacturers’ protocol and using 100 ng of the linear PCR product. The resulting bacterial colonies were subjected to colony PCR (Supplementary Table S3) and analysed by *ScaI* restriction digestion of the colony PCR products (see Supplementary Fig. S3 and Table S4).

## Additional Information

**How to cite this article**: Trehan, A. *et al.* REPLACR-mutagenesis, a one-step method for site-directed mutagenesis by recombineering. *Sci. Rep.* **6**, 19121; doi: 10.1038/srep19121 (2016).

## Supplementary Material

Supplementary Data

## Figures and Tables

**Table 1:** The mutations in the plasmids were introduced by REPLACR-mutagenesis and were verified by DNA sequencing.

Mutation	Colonies Screened	Correct colonies	Efficiency (%)
LHCGR_Asn291Ser	5	4	80
LHCGR_Val454Ile	5	5	100
FSHR_Ala444Thr	8	6	75
FSHR_Gly70Ala	8	5	63
β2AR_Asp79Asn	5	1	20
β2AR_Asp130Asn	5	4	80
β2AR_Cys341Gly	5	1	20
β2AR_Tyr350Ala	5	5	100
LHCGR_1850delG	8	7	88
LHCGR_Lys12-Leu15del	8	5	63
LHCGR_deletion_144kb (RPCI-11-186L7)	8	1	13
LHCGR_Leu10-Gln17Dup	16	2	13
CRY2_NLS (45 nt addition)	8	6	75
Flexible domain (60 nt addition)	8	6	75
*Wnt1* targeting vector	8	4	50

The number of bacterial colonies screened and the number of correct mutations found over background were used to calculate the associated efficiencies. The median efficiency associated with all the mutations combined was 75%.
