# Supplementary material for: REPLACR-mutagenesis, a one-step method for site-directed mutagenesis by recombineering
Source: Sci Rep. 2016 Jan 11;6:19121. doi: 10.1038/srep19121 (PMC4707547; doi:10.1038/srep19121)
Supplement: Supplementary Data [file srep19121-s1.pdf]

Supplementary Data

REPLACR-mutagenesis, a one-step method for site-directed mutagenesis by recombineering

Ashutosh Trehan [1], Michał Kiełbus [4], Jakub Czapinski [4], Andrzej Stepulak [4], Ilpo Huhtaniemi [1,2] and Adolfo Rivero-Müller [1,3,4*]

[1] Department of Physiology, Institute of Biomedicine, University of Turku, Turku, Finland

[2] Department of Surgery and Cancer, Institute of Reproductive and Developmental Biology, Hammersmith Campus, Imperial College London, London, United Kingdom

[3] Faculty of Natural Sciences and Technology, Åbo Akademi University, Turku, Finland

[4] Department of Biochemistry and Molecular Biology, Medical University of Lublin, Lublin, Poland.

*Corresponding author

Email: adoriv@utu.fi (ARM)

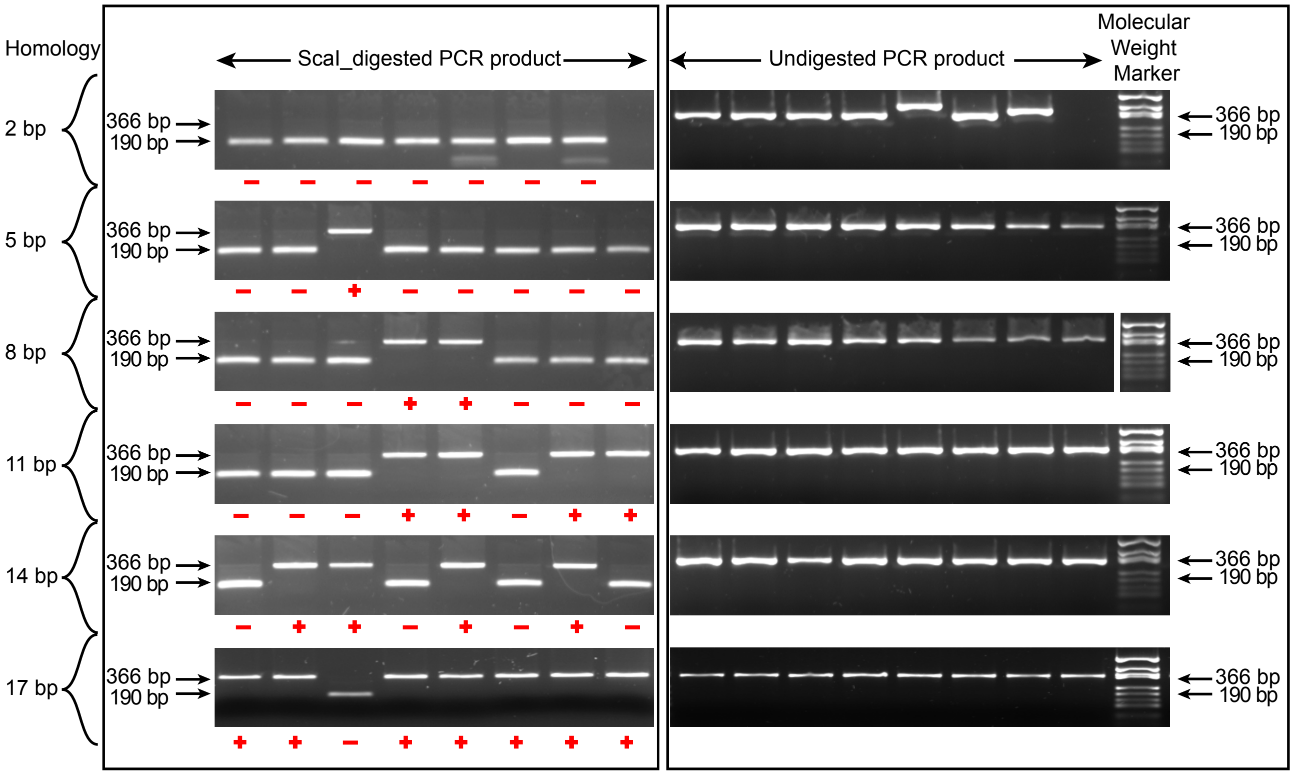

Supplementary Figure S1. PCR products with homology at their termini ranging from 2 bp to 17 bp were transformed in Red/ET bacteria and the resulting bacterial colonies were picked for colony PCR. The mutated plasmids should yield 366 bp long PCR products (not digested by *ScaI*), whereas the wild-type (WT) background should yield 364 bp long PCR products which upon *ScaI* digestion gives 190 bp and 174 bp products (indistiguishable as a single band). The mutated plasmids are marked as "+" while the LHCGR_WT background is marked as "-". The data is presented for one representative experiment with three independent repeats.

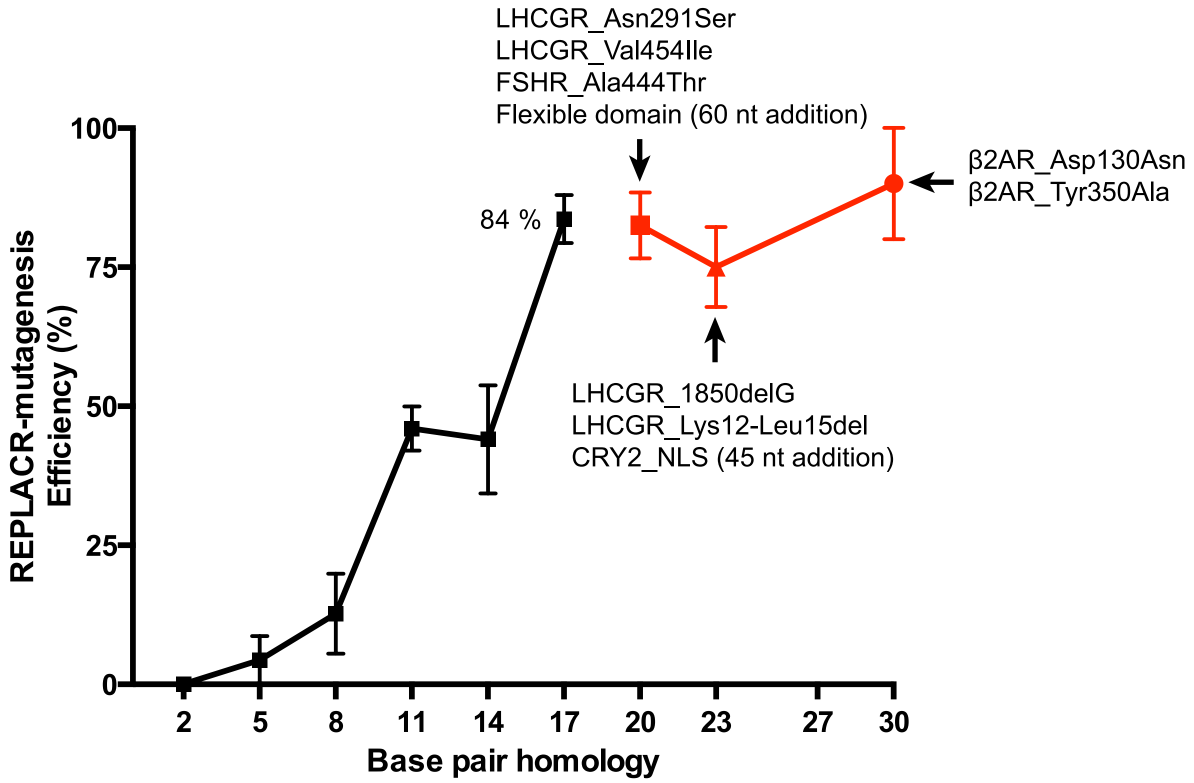

Supplementary Figure S2. Mutants made using homology longer than 17 bp (shown in red) by REPLACR-mutagenesis show very similar efficiencies to those made using a 17 bp homology (mean ± SEM), thereby demostrating that a 17 bp homology at the ends of PCR termini is sufficient for mutagenesis. The mutants with 20, 23 and 30 bp homology are marked with an arrow and their efficiencies are mentioned in Table 1. The data for 2 bp to 17 bp homology is the same as mentioned in Figure 2a.

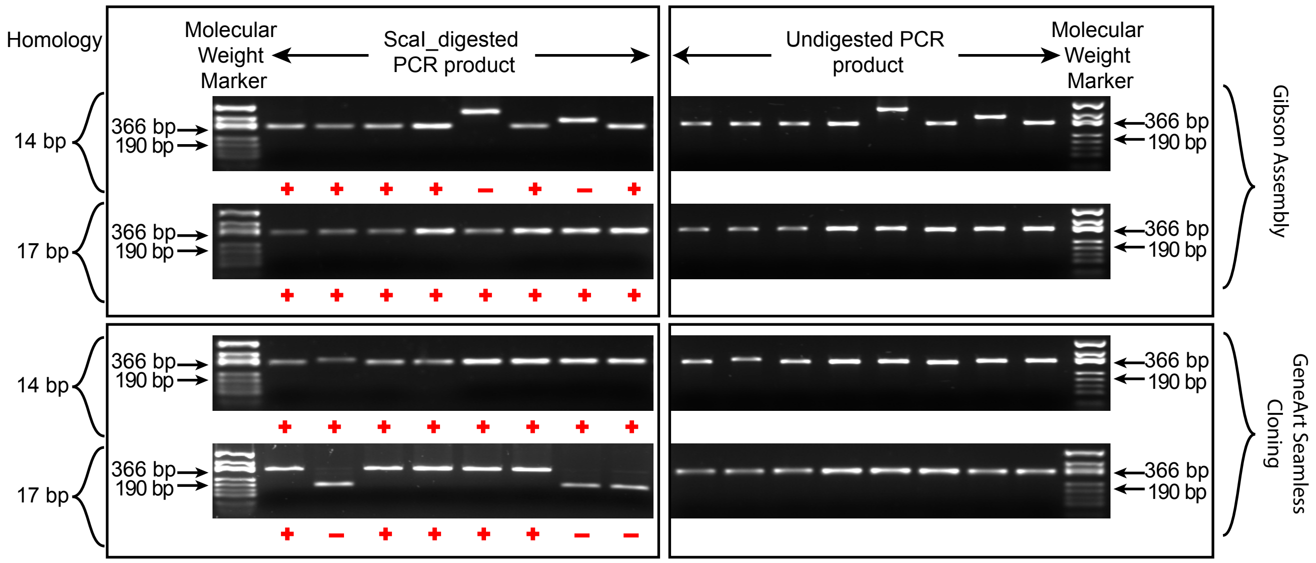

Supplementary Figure S3. PCR products with 14 bp and 17 bp homology at their ends were used for recombination using Gibson asembly and GeneArt Seamless cloning commercial kits. The resulting bacterial colonies containing circular plasmids were used for colony PCR. The expected PCR products (366 bp for plasmids with mutated *ScaI* site and 364 bp for LHCGR_WT background with intact *ScaI* site) were subjected to *ScaI* digestion. The PCR products from mutated plasmids (366bp) are not digested (marked as "+") while LHCGR_WT background results in 190 bp and 174 bp products (seen as a single band and marked as "-"). The presented data is for one representative experiment that has been independently repeated three times.

a. LHCGR_Asn291Ser mutation [A —> G]

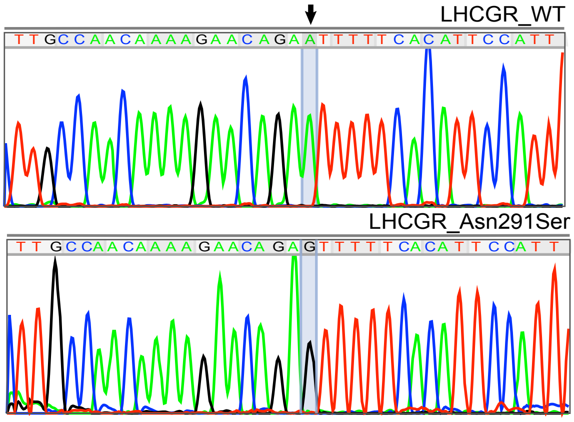

b. LHCGR_Val454Ile mutation [G —> A].

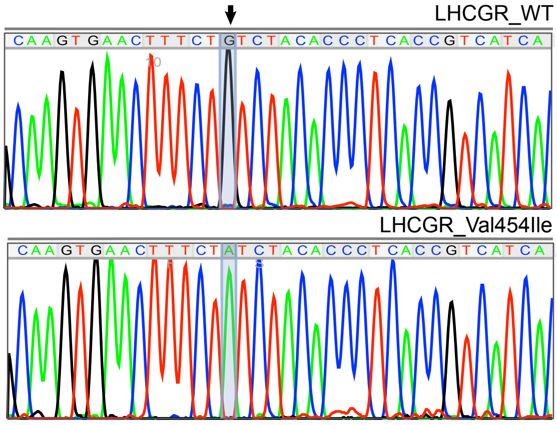

c. FSHR_Ala444Thr mutation [G —> A]

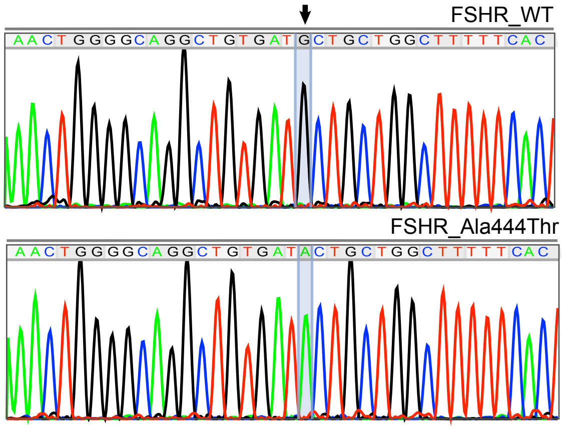

d. FSHR_Gly70Ala mutation [G —> C]

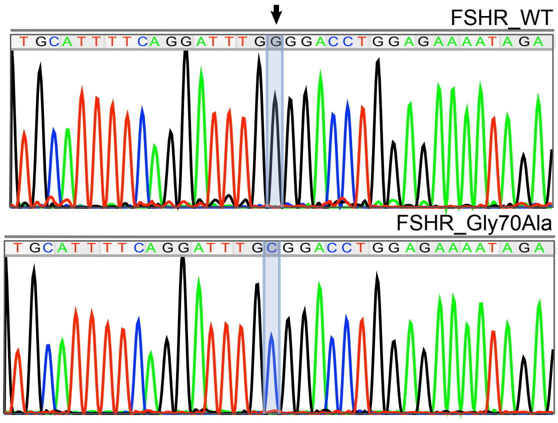

e. β2AR_Asp79Asn mutation [G —> A]

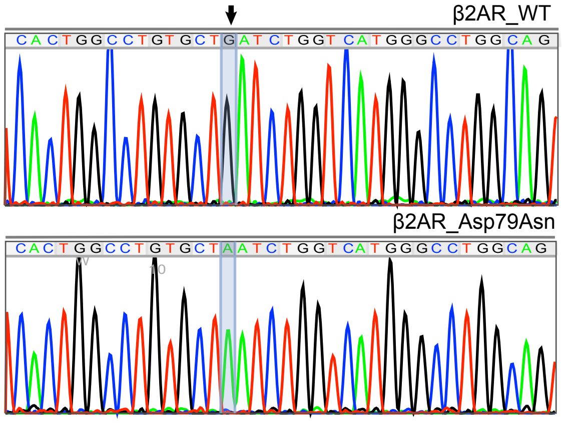

f. β2AR_Asp130Asn mutation [G —> A]

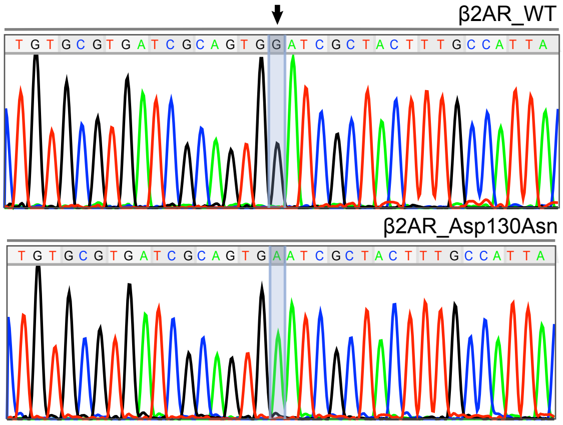

g. β2AR_Cys341Gly mutation [T —> G]

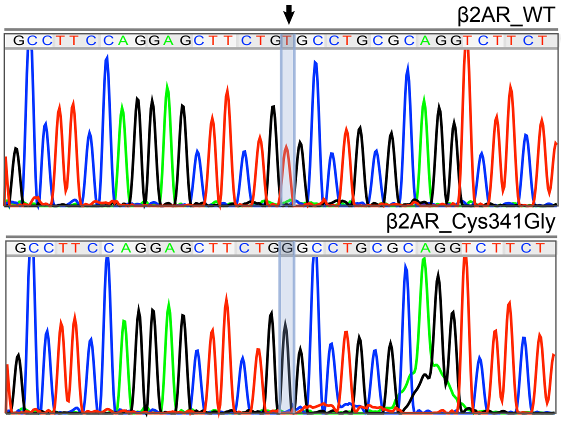

h. β2AR_Tyr350Ala mutation [TA —> GC]

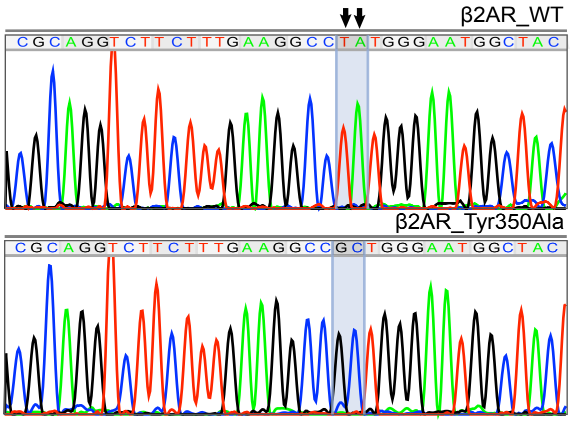

Supplementary Figure S4. Point substitutions made using REPLACR-mutagenesis. The nucleotides changed are marked with an arrow and are highlighted. The original wild type (WT) sequences are on top and the mutated sequences in the bottom row, with gene name followed by the associated mutation.

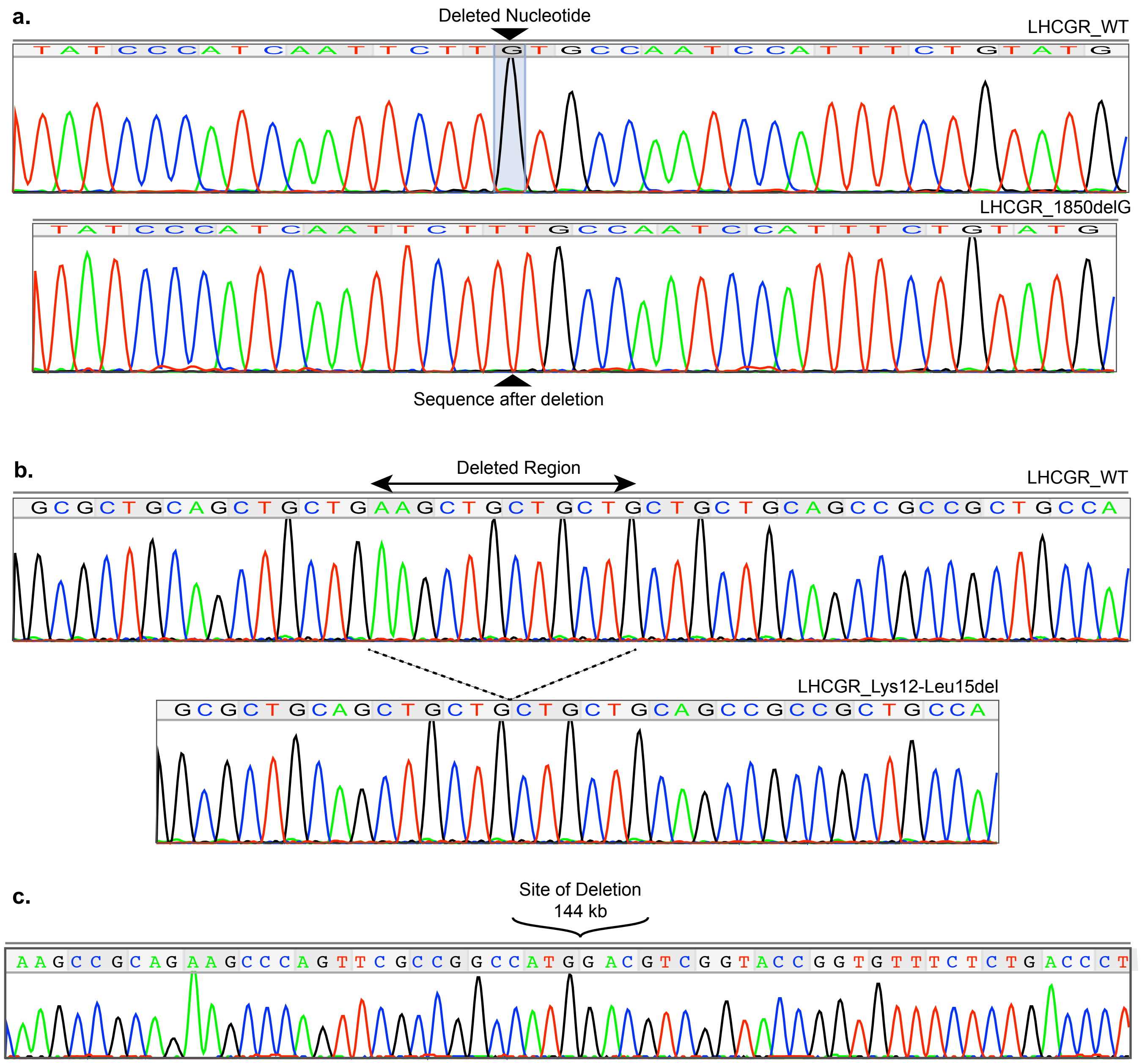

Supplementary Figure S5. Deletion of nucleotides by REPLACR-mutagenesis.
(a) Deletion of one nucleotide [G] in LHCGR_WT to create LHCGR_1850delG mutant. **(b)** Deletion of 12 nucleotides in LHCGR_WT for creating LHCGR_Lys12-Leu15del mutant. **(c)** A 144 kb DNA sequence was deleted from a human LHCGR BAC clone (RPCI-11-186L7) and the resulting circularized vector was verified for the correct recombination site by sequencing. The site of deletion is marked in the chromatogram.

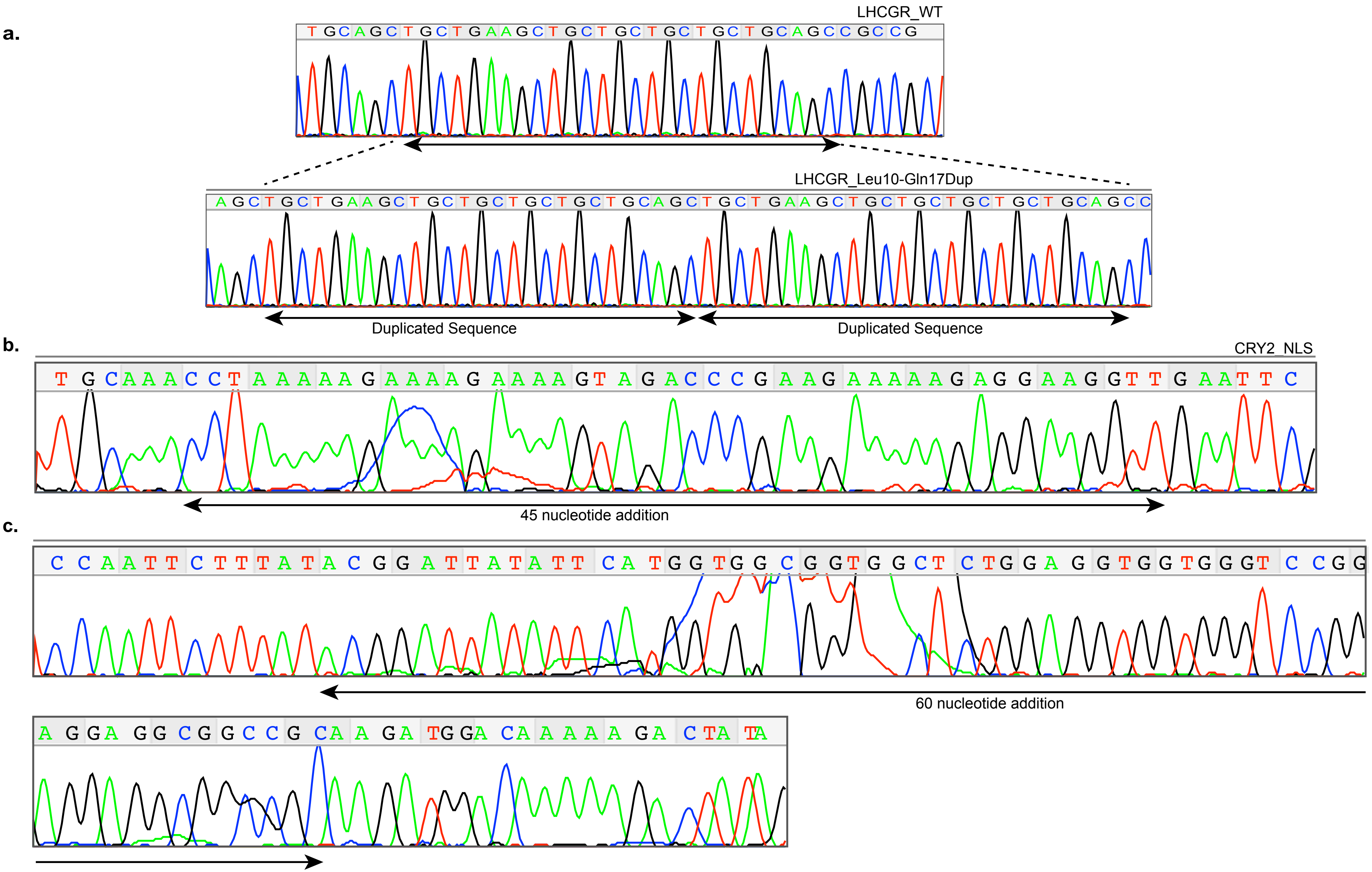

Supplementary Figure S6. Additions using REPLACR-mutagenesis.
(a) Duplication/addition of 27 nucleotides in wild type (WT) *LHCGR* to create LHCGR_Leu10-Gln17Dup mutant. **(b)** Addition of 45 nucleotides (CCTAAAAAGAAAAGAAAAGTAGACCCGAAGAAAAAGAGGAAGGTT) containing a nuclear localization signal (NLS) to the *CRY2* gene. **(c)** 60 nucleotide addition of a flexible domain (ACGGATTATATTCATGGTGGCGGTGGCTCTGGAGGTGGTGGGTCCGGAGGAGGCGGCCGC) to *CRY2* gene.

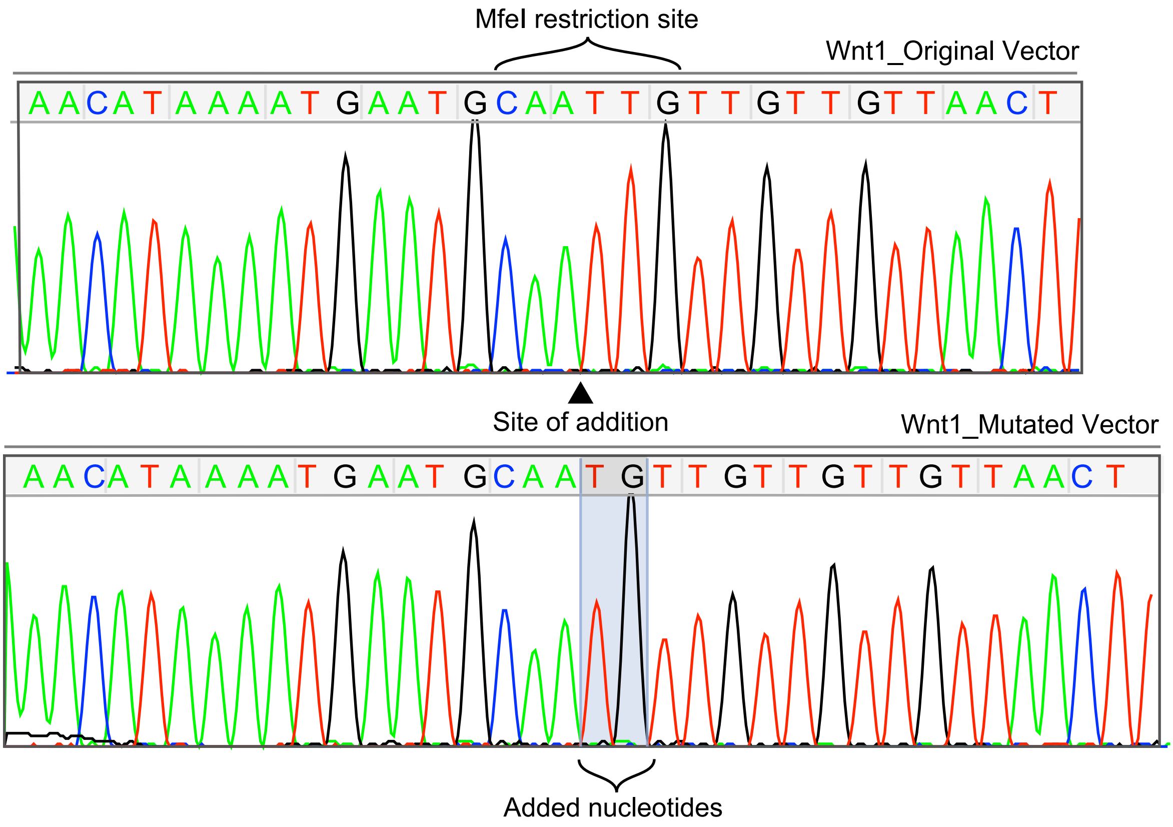

Supplementary Figure S7. Addition of two nucleotides (TG) in the middle of *MfeI* restriction site (CAATTG, marked with triangle; upper row) in the original *Wnt1* targeting vector to generate a mutated *Wnt1* targeting vector (CAA<u>TG</u>TTG).

Deleted Sequence:

AATTAGTTGTTTTCAAAGCAAATGAACTAGCGATTAGTCGCTATGACTTAACGGAGCATGAAACCAAGCTAATTTTATGCTGTGTGGCACT
ACTCAACCCCACGATTGAAAACCCTACAAGGAAAGAACGGACGGTATCGTTCACTTATAACCAATACGCTCAGATGATGAACATCAGTAG
GGAAAATGCTTATGGTGTATTAGCTAAAGCAACCAGAGAGCTGATGACGAGAACTGTGGAAATCAGGAATCCTTTGGTTAAAGGCTTTGA
GATTTTCCAGTGGACAAACTATGCCAAGTTCTCAAGCGAAAAATTAGAATTAGTTTTTAGTGAAGAGATATTGCCTTATCTTTTCCAGTTAA
AAAAATTCATAAAATATAATCTGGAACATGTTAAGTCTTTTGAAAACAAATACTCTATGAGGATTTATGAGTGGTTATTAAAAGAACTAACAC
AAAAGAAAACTCACAAGGCAAATATAGAGATTAGCCTTGATGAATTTAAGTTCATGTTAATGCTTGAAAATAACTACCATGAGTTTAAAAGG
CTTAACCAATGGGTTTTGAAACCAATAAGTAAAGATTTAAACACTTACAGCAATATGAAATTGGTGGTTGATAAGCGAGGCCGCCCGACT
GATACGTTGATTTTCCAAGTTGAACTAGATAGACAAATGGATCTCGTAACCGAACTTGAGAACAACCAGATAAAAATGAATGGTGACAAAA
TACCAACAACCATTACATCAGATTCCTACCTACGTAACGGACTAAGAAAAACACTACACGATGCTTTAACTGCAAAAATTCAGCTCACCAG
TTTTGAGGCAAAATTTTTGAGTGACATGCAAAGTAAGCATGATCTCAATGGTTCGTTCTCATGGCTCACGCAAAAACAACGAACCACACT
AGAGAACATACTGGCTAAATACGGAAGGATCTGA

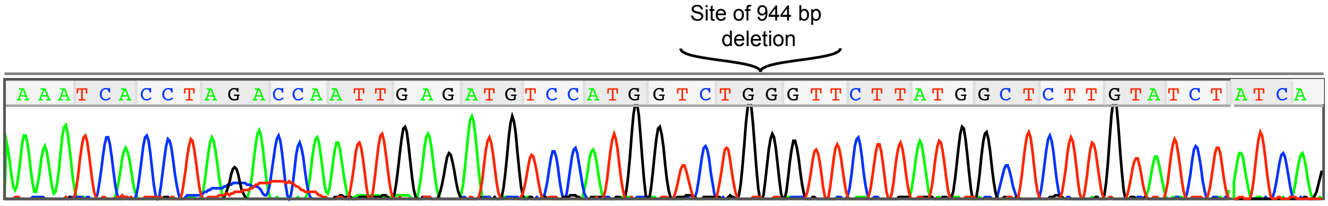

Supplementary Figure S8. A 944 bp DNA sequence encoding RepA protein (temperature sensitive repressor) was deleted (sequence specified in rectangular box) in Red/ET plasmid using REPLACR-mutagenesis. The site of deletion is marked in the chromatogram and the resulting sequence was correct, as expected.

Supplementary Table S1. Primers used to determine the length of optimal base pair (bp) homology in REPLACR-mutagenesis. The homologous base pairs are highlighted in bold. All primers are 23 bp in length.

Homology (bp)	Forward primer (5'-3')	Reverse primer (5'-3')
2	**AT**ACTATAACCATGCCATAGACT	**AT**ACTGGCCCTTGGTTTGGGAAT
5	**ATACT**ATAACCATGCCATAGACT	**AGTAT**ACTGGCCCTTGGTTTGGG
8	**ATACTATA**ACCATGCCATAGACT	**TATAGTAT**ACTGGCCCTTGGTTT
11	**ATACTATAACC**ATGCCATAGACT	**GGTTATAGTAT**ACTGGCCCTTGG
14	**ATACTATAACCATG**CCATAGACT	**CATGGTTATAGTAT**ACTGGCCCT
17	**ATACTATAACCATGCCAT**AGACT	**TGGCATGGTTATAGTAT**ACTGGC
20	**ATACTATAACCATGCCATAG**ACT	**CTATGGCATGGTTATAGTAT**ACT
23	**ATACTATAACCATGCCATAGACT**	**AGTCTATGGCATGGTTATAGTAT**

Supplementary Table S2. PCR products with homology ends ranging from 2 bp to 17 bp after REPLACR-mutagenesis resulted in below-mentioned efficiencies. The data is presented for one experiment, with three independent repeats.

Homology (bp)	Colonies Obtained	Colonies Screened	Correct Colonies	Efficiency (%)
2	13	7	0	0
5	77	8	1	13
8	>100	8	2	25
11	>100	8	4	50
14	>100	8	4	50
17	>100	8	7	88

Supplementary Table S3. Colony PCR conditions for bacterial colonies obtained using PCR products with 2-17 bp homology.

	Temperature	Time
Initial denaturation	94 °C	5 min
Denaturation	98 °C	30 s
Annealing	50 °C	30 s
Extension	72 °C	1 min (30 cycles)
Final extension	72 °C	5 min

Supplementary Table S4. Efficiency comparison of REPLACR-mutagenesis with commercial kits (Gibson Assembly and GeneArt Seamless cloning). PCR products with 14 bp homology at their termini results in lowest efficiency for REPLACR mutagenesis among the methods compared whereas PCR products with 17 bp homology yields similar efficiencies for all the methods. The presented table is for one representative experiment only, with three independent repeats.

Homology	Mutagenesis method	Colonies obtained	Colonies screened	Correct colonies	Efficiency (%)
14 bp	Gibson assembly	>100	8	6	75
	GeneArt Seamless Cloning	85	8	8	100
	REPLACR-mutagenesis	>100	8	4	50
17 bp	Gibson assembly	>100	8	8	100
	GeneArt Seamless Cloning	21	8	5	63
	REPLACR-mutagenesis	>100	8	7	88

Supplementary Table S5. Primers used in this study. Mutated nucleotides in the original plasmids are shown in bold and the homology regions are underlined.

Gene and Mutation	Forward (5´-3´)	Reverse (5´-3´)
LHCGR_Asn291Ser	CAAAAGAACAGA**G**TTTTTCACATTCCATTTCTGAA	AGAAATGGAATGTGAAAAA**C**
LHCGR_Val454Ile	TATTCGCAAGTGAACTTTCT**A**TCTACACCCTCACCGTCATCAC	ATGACGGTGAGGGTGTAGA**T**
FSHR_Ala444Thr	CTGGCAAACTGGGGCAGGCTGTGAT**A**CTGCTGGCTTTTTCACTGTCTTTG	ACAGTGAAAAAGCCAGCAG**T**
FSHR_Gly70Ala	ATTTTCAGGATTTGCGGAC**C**TGGAGAAAATAGAGATC	TCTCTATTTTCTCCAGGTCC**G**
β2AR_Asp79Asn	TGTGCT**A**ATCTGGTCATGGGCCTGGCAGTGGTGC	CACTGCCAGGCCCATGACCAGAT**T**AGCACAGGCCAG
β2AR_Asp130Asn	GCAGTG**A**ATCGCTACTTTGCCATTACTTC	TGAAGTAATGGCAAAGTAGCGAT**T**CACTGCGATCAC

	ACCTTTC	
β2AR_Cys341Gly	CTTCTGGGCCTGCGCAGGTCTTCTTTGAAGGCCTAT	CTTCAAAGAAGACCTGCGCAGGCCCAGAAGCTCCTG
β2AR_Tyr350Ala	AAGGCCGCTGGGAATGGCTACTCCAGCAACGGCAAC	GTTGCTGGAGTAGCCATTCCCAGCGGCCTTCAAAGA
LHCGR_1850delG	ATCCCATCAATTCTTTGCCAATCCATTTCTGTATGCA	TGCATACAGAAATGGATTGGCAA
LHCGR_Lys12-Leu15del	CAGCTGCTGCTGCTGCAGCCGCCG	GGCGGCTGCAGCAGCAGCAGCTGCAGCGCCGAGAAC
LHCGR_deletion_144kb (RPCI-11-186L7)	ATGGACGTCGGTACCGGTGTTTCTCTGACCCTGTTTC	CCGGTACCGACGTCCATGGCCGGCGAACTGGGCTTCT
LHCGR_Leu10-Gln17Dup	**TGCTGCTGCTGCAGC**CGCCGCTGCCACGAGCGTAC	**GCTGCAGCAGCAGCAGCAGCTTCAGCA**GCTGCAGCAGCAGCAGCAGCTTCAGCA
CRY2_NLS (45 nt addition)	AAGAAAAGTAGACCCGAAGAAAAAGAGGAAGGTTGAATTCGGCAGTGGAGAGGGCA	TTTTCTTCGGGTCTACTTTTCTTTTCTTTTTAGGTTTGCAACCATTTTTTCCCAAA
Flexible domain (60 nt addition)	CGGTGGCTCTGGAGGTGGTGGGTCCGGAGGAGGCGGCCGCAAGATGGACAAAAAGACTATAGTT	CACCACCTCCAGAGCCACCGCCACCATGAATATAATCCGTATAAAGAATTGGAGCGTAAGTCTG
Wnt1 targeting vector	**TG**TTGTTGTTGTTAACTTGTTTATTGCAGCTTATAATG	AGTTAACAACAACAA**CA**TTGCATTCATTTTATGTTTC
ET/Red Deletion_944bp	GATGT**CC**ATG**G**TCTGGGTTCTTATGGCTCTTGTATCT	CAGA**C**CAT**GG**ACATCTCAATTGGTCTAGGTGA

Supplementary Table S6. Sequencing primers for checking the final mutant plasmids.

Gene and Mutation	Forward Sequencing primer (5′-3′)	Reverse Sequencing Primer (5′-3′)
• LHCGR_Asn291Ser<br>• LHCGR_Leu10-Gln17Dup<br>• LHCGR_Lys12-Leu15del	ATATAAATTCTGGCTGGCGTGG	TGAAAGCTTGAGATGGGATCAC

• LHCGR_1850delG	AATTGCTATGTTGCCCCTTG	GCCTACATACCTCGCTCTGC
• LHCGR_Val454Ile	GCTAATTGCCACGTCATCCT	TGGGGAAGCAAATACTGACC
• FSHR_Gly70Ala	TACAAAGATGATGATGATAAG	CTCAGAGATTTGCCGTCTCC
• FSHR_Ala444Thr	AGGCTAGGGGTCAGAGATCC	ACCTTGAGGGAGGCAGAAAT
• β2AR_Asp79Asn<br>• β2AR_Asp130Asn	CTCTCATCGTCCTGGCCATC	ATGATCACCCGGGCCTTATT
• β2AR_Cys341Gly<br>• β2AR_Tyr350Ala	GCCCAGATTTCAGGATTGCC	GATGGCCCACAAAGTCTTCC
• LHCGR_deletion_144 kb (RPCI-11-186L7)	GCAGATGGTAGGGGACAAGA	GCTATGTGGCTGTTGGGATT
• *Wnt1* targeting vector	CGGTCGCTACCATTACCAGT	GTGGACATCTCTTGGGCACT
• ET/Red Deletion_944bp	AGCGGAATTTACAGAGGGTCT	AGGCTGTCTATGTGTGACTGT

Supplementary Table S7. PCR conditions for deletion of 144 kb from human LHCGR BAC (RPCI-11-186L7).

	**Temperature**	**Time**
Polymerase activation	94 °C	2 min
Denaturation	98 °C	30 s
Annealing	54 °C	30 s
Extension	68 °C	36 min (20 cycles)

Supplementary Table S8. PCR conditions for *Wnt1* targeting plasmid

	**Temperature**	**Time**
Polymerase activation	94 °C	2 min
Denaturation	98 °C	10 s
Annealing	62 °C	30 s
Extension	68 °C	27 min (20 cycles)

Supplementary Table S9. PCR conditions for deletion of 944 bp from Red/ET plasmid

	**Temperature**	**Time**
Polymerase activation	94 °C	2 min
Denaturation	98 °C	10 s
Annealing	61 °C	30 s
Extension	68 °C	10 min (25 cycles)

Supplementary Table S10. PCR conditions for β2AR_Asp130Asn

	**Temperature**	**Time**
Polymerase activation	94 °C	2 min
Denaturation	98 °C	10 s
Annealing	64 °C	30 s
Extension	68 °C	8 min (30 cycles)

Supplementary Table S11. PCR conditions for β2AR_Asp79Asn, β2AR_Cys341Gly and β2AR_Tyr350Ala

	**Temperature**	**Time**
Polymerase activation	94 °C	2 min
Denaturation	98 °C	10 s
Annealing/ Extension	70 °C	8 min (5 cycles)
Denaturation	98 °C	10 s
Annealing/ Extension	68 °C	8 min (5 cycles)
Denaturation	98 °C	10 s
Annealing	66 °C	30 s
Extension	68 °C	8 min (20 cycles)

Supplementary Table S12. PCR conditions for all other mutants

	**Temperature**	**Time**
Polymerase activation	98 °C	2 min
Denaturation	98 °C	10 s
Annealing	56 °C	30 s
Extension	68 °C	1 min/Kb (30 cycles)
